# Left atrial spindle cell sarcoma: A case report and literature review

**DOI:** 10.1097/MD.0000000000024044

**Published:** 2021-01-15

**Authors:** Jin Qin, Rui Li, Fei Ma, Haojie Li, Zemin Fang, Yujie Fei

**Affiliations:** a Division of Cardiology, Department of Internal Medicine; b Department of Radiology; c Department of Cardiothoracic and Vascular Surgery, Tongji Hospital, Tongji Medical College, Huazhong University of Science and Technology, Wuhan, P. R. China.

**Keywords:** cardiac autotransplantation, cardiac magnetic resonance, contrast-enhanced echocardiography, polychemotherapy, primary spindle cell sarcoma

## Abstract

**Rationale:**

Cardiac primary spindle cell sarcoma is 1 of the rarest cardiac malignancies, with only a few cases reported so far. Herein, we reported a case of left atrial spindle cell sarcoma diagnosed and treated by a multidisciplinary approach, and retrospectively reviewed other reported cases.

**Patient concerns:**

A 49-year-old woman presented to our hospital with 2 weeks of gradual onset of dyspnea on exertion, dry cough and subacute fever.

**Diagnosis:**

The patient was initially revealed a left atrium mass by 2-dimensional transthoracic echocardiography. Based on the contrast-enhanced echocardiography and cardiac magnetic resonance imaging, she was subsequently suggested to have a cardiac malignant tumor. And the post-operative histopathology confirmed the tumor to be a cardiac primary spindle cell sarcoma.

**Interventions:**

The tumor was completely resected using autotransplantation. The patient was referred for polychemotherapy afterwards.

**Outcomes:**

Our patient underwent the tumor resection, with subsequent adjuvant polychemotherapy, and the tumor has not recurred during 12 months of follow-up.

**Lessons:**

Due to the rarity of these tumors and nonspecific symptoms, they are often difficult to diagnose preoperatively and missed occasionally. Thus, improving our understanding of the disease and facilitating its early diagnosis are essential.

## Abstract 2

Supplemental Digital Content is available in the text

## 1 Introduction

Primary cardiac tumors are rare with an incidence ranging from 0.017 to 0.019. Depending on the exact location, they can present with a variety of cardiopulmonary symptoms, such as chest discomfort, dyspnea, syncope, heart failure, pericardial effusion and tamponade, valvular abnormalities or conduction abnormalities. Twenty-5 percent of the cardiac tumors are malignant with 95% of these being reported as sarcomas. Among them, the least reported cardiac tumors are spindle cell sarcomas, which are clinically aggressive neoplasms associated with a poor prognosis.^[1,2]^ Because of nonspecificity of symptoms and rarity of these tumors, they are often difficult to diagnose preoperatively and missed occasionally. The advent of modern investigative tools including contrast-enhanced ultrasound imaging, computed tomography (CT) scan and cardiac magnetic resonance (CMR) increases the likelihood of preoperative diagnosis. This is illustrated in the presented case report of left atrial spindle cell sarcoma diagnosed and treated by a multidisciplinary approach in our hospital with a brief review of current literature.

### 1.1 Consent statement

The present study was approved by the Ethics Committees of Tongji Hospital. Written informed consent was obtained from the patient for the publication of this case report.

## 2 Case report

A 49-year-old woman with no medical history presented to our hospital with 2 weeks of gradual onset of dyspnea on exertion, dry cough and subacute fever. She denied any recent illnesses, or unintentional weight loss. She was not taking any medications or tobacco, alcohol, or illicit drugs.

On physical examination, her blood pressure was 93/59 mm Hg, heart rate was 124 bpm, her regular, respiratory rate was 23 breaths per minute, her temperature was 40°C, and her oxygen saturation was 95% (ambient air). Her cardiovascular, respiratory, abdominal and neurological examinations were unremarkable. Laboratory findings showed the inflammatory markers, such as white blood cell and C-reactive protein, were also within the normal range. The patient had low serum albumin and high globulin, with elevated lactic dehydrogenase, but the results of the remainder of liver function, kidney function and coagulation studies were almost normal. She was anemic, with hemoglobin of 7.2 g/dL. Her tumor markers (carcinoembryonic antigen, cancer antigen 19–9, cancer antigen 125, ɑ-fetoprotein, and human chorionic gonadotropin) were not elevated; human immuno-deficiency virus was non-reactive.

**Figure 1:**
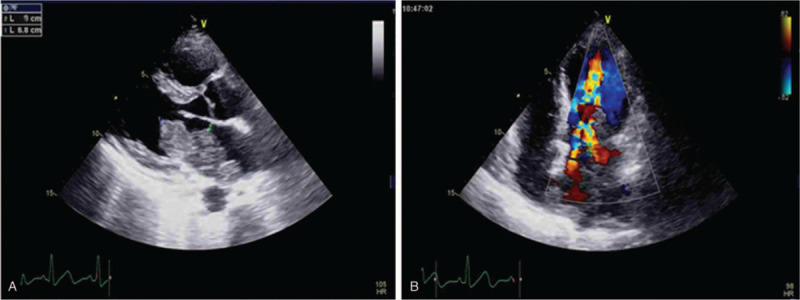
(A) Cardiac image section 4 cavities of an echocardiography: a large, irregular mass located on the lateral wall of the left atrium; (B) Color Doppler flow imaging (CDFI) shows a multicolored high-speed blood flow signal during diastole as a typical sign for mitral stenosis.

Her electrocardiogram showed sinus tachycardia with no significant ST-T abnormalities. Transthoracic echocardiography (TTE) revealed a large, irregular, solitary mass, measuring 6.8 × 3.0 cm, located on the lateral wall of the left atrium, close to the inter-atrial septum. It was broad based, occupying nearly the entire left atrium and was mobile, which prolapsed into the left ventricle during diastole (Supplementary Videos 1 {Video that demonstrates the dynamic imaging of the parasternal 4 chamber view by the 2-dimensional TTE, 1 second, 0.95 MB, http://links.lww.com/MD/F506} and 2 {Video that demonstrates the dynamic imaging of the apical 4 chamber view by the 2-dimensional TTE, 1 second, 0.99 MB, http://links.lww.com/MD/F507}, which demonstrates the dynamic imaging of the parasternal and apical 4 chamber views by the 2-dimensional TTE). A zoomed image of the apical 4-chamber view is shown in Fig. 1A. The left ventricular ejection fraction was 70%. There was mild mitral stenosis (Fig. 1B). Pulmonary systolic artery pressure was 49 mm Hg. There was no pericardial effusion. Afterwards, the examination continued with a contrast-enhanced echocardiography, which helped exclude a thrombus and define the vascularity of the cardiac mass. After obtaining informed consent from the patient, 2.4 mL of the sulfur hexafluoride microbubble contrast agent SonoVue (Bracco, Geneva, Switzerland) was administered intravenously with sodium chloride at constant rate (approximately 1 mL/min), which kept the contrast dissolved during the infusion. After injection of the contrast agent, the mass was rapidly filled with echocardiographic contrast, indicating an intense vascularization and excluding thrombus (Supplementary Video 3 {Video that demonstrates an intense vascularization of the left atrial mass after injected SonoVue (ultrasound contrast agents), 11 seconds, 4.70 MB, http://links.lww.com/MD/F508}, which demonstrates the dynamic imaging of the left atrial mass after injected SonoVue). Additional images with a destruction-replenishment technique were acquired, using transient high-mechanical Index flash imaging to deplete microbubbles and observe subsequent replenishment over the following cardiac cycles (normal resting myocardial contrast replenishment should occur within 4 to 5 seconds at a normal resting heart). As illustrated in Video 4 (Supplementary Video 4 {Video that demonstrates a quick contrast re-filling of the left atrial mass used the contrast-assisted destruction-replenishment technique, 11 seconds, 4.69 MB, http://links.lww.com/MD/F509}, which demonstrates the dynamic imaging of the left atrial mass used the contrast-assisted destruction-replenishment technique), quick contrast re-filling of the mass was observed also suggestive of high vascularization of the tumor mass, typical of malign tumors.

**Figure 2:**
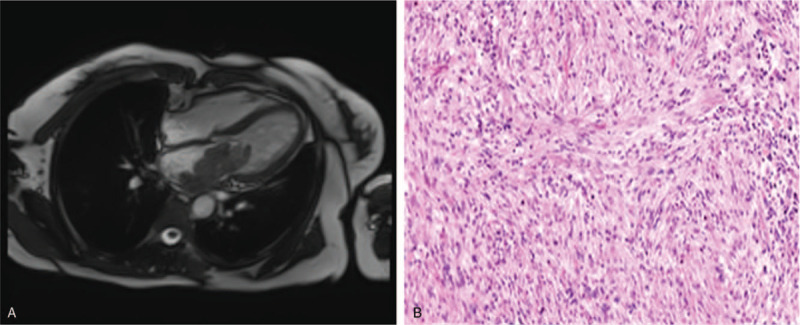
(A) CMR: the big mass prolapsed from the left atrium into the left ventricle in diastole; (B) Pathological results confirmed cardiac spindle cell sarcomas (original × 100).

In order to better investigate the disease, a CMR was performed. The study showed again a big mass in the left atrium. The mass, measuring 4.9 × 2.9 cm, was seen prolapsing through the mitral valve into the left ventricle in diastole. Of note, the tumor was widely attached to the lateral wall of the left atrium (Fig. 2A). The mass was isointense in T1-weighted imaging and hyperintense on T2-weighted imaging. During first-pass perfusion imaging, progressive contrast uptake was observed in the mass. Delayed contrast-enhanced imaging revealed heterogeneous hyperenhancement of the mass.

The results of echocardiography and CMR suggested that tumors could be malignant. Then, a thoracic abdominopelvic CT was done which ruled out extracardiac tumor involvement. CT of the brain was negative for metastases. The cardiac surgery was decided to perform. The patient underwent surgery for excision of the left atrial mass via a median sternotomy. The mass was very vascularized and had a broad base originated from the lateral wall of the left atrium in surgery. Cardiac autotransplantation (cardiac explantation, *ex vivo* tumor resection, reconstruction, and reimplantation) was then performed. Finally, the cardiac mass was removed completely, about 4 × 5 cm with a moderate texture. Immunohistochemical tests found only focal reactivity to CD34 and INI1, negative for the following markers: EMA, CD117, SMA, Desmin,caldesmon, S100, STAT6, Myogenin, FLI1, Calretinin, and CK5/6. The measured proliferation index with Ki-67 was 40%, conclusive of high-grade spindle cell sarcoma (Fig. 2B). The patient was subsequently referred to the department of oncology for chemotherapy treatment. Follow-up color Doppler echocardiography scans and CMR obtained from 12 months after the operation showed no recurrent mass.

## 3 Discussion

Cardiac primary spindle cell sarcoma is 1 of the rarest cardiac malignancies. These tumors are highly aggressive, infiltrating all the layers of the heart and metastasize rapidly, reported mean survival of approximately 3 months to 1 year. Cardiac spindle cell sarcomas are tumors of mesenchymal origin, more commonly affecting the large blood vessels. They are extremely rare in the heart, with only a few cases reported so far. A summary of other cases reported previously and our case are shown in Table 1.^[3–9]^ The tumor usually infiltrates the left atrium, as seen in our patient. Lung metastases (40%) and extrathoracic metastasis (20%) to kidneys, lymph nodes, brain, and skin are often seen. Tumor emboli are common, causing distant metastases involving bone, peritoneum, liver, and mesenteric lymph nodes.^[10,11]^ In our case, the patient was first admitted to our hospital with fever of unknown origins. Due to a mass lesion involving the left atrium observed by TTE, she was transferred to the cardiovascular medicine department for further evaluation.

**Table 1:** Literature review of cardiac spindle cell sarcomas and our case.

Case / Refs.	Age (yr) / Sex	Clinical presentation	Size / site	Imaging Modalities	Treatment	Follow up
1 /^[3]^	47 / Male	Dyspnoea, heaviness in the chest	5 × 3 × 3 cm / LA	TTE, CT	Partial resection	9 mo / Died
2 /^[4]^	46 / Male	Shortness of breath, diminished exercise tolerance	8 × 4.5 × 3 cm / LA	TTE, TEE, CT, MRI	Partial resection followed chemotherapy	12 mo / Recurrence
3 /^[5]^	69 / Male	Abdominal discomfort, weight loss	4.3 × 4.5 × 2.9 cm / LA	TTE, TEE, CT, MRI	Extensive surgical resection	10 d / Died
4 /^[6]^	28 / Male	Chest pain, cough, fever and weight loss	Unknown size / RV	TTE, TEE, CT	Resection followed chemotherapy	3 mo / No recurrence
5 /^[7]^	42 / Male	Dyspnea, diminished exercise tolerance	Unknown size / LA	TTE, CT, MRI	Resection	8 mo / Recurrence
6 /^[8]^	34 / Female	Hematemesis, back pain, weight loss	7.2 × 3.7 cm / LA	TTE, TEE, CT, MRI	Unresectable and biopsy taken	7 mo / Died
7 /^[9]^	57/ Female	No symptoms	Unknown size / LA	TTE, TEE, CMR, PET-CT	Partial resection	11 mo / Died
8 (Our case)	49/Female	Dyspnea, dry cough, fever	6.8 × 3.0 cm / LA	TTE, CT, CMR	Resection followed adjuvant treatment	12 mo / No recurrence

CMR = cardiac magnetic resonance, CT = computed tomography, LA = left atrium, MRI = Magnetic resonance imaging, PET-CT = positron emission tomography- Computed, TEE = transesophageal echocardiography, TTE = transthoracic echocardiogrphy.

TTE is the first-line imaging modality that usually identifies a cardiac mass, providing important information including location, size, morphology, mobility, and hemodynamic effect. According to American Society of Echocardiography guidelines, complete TTE is recommended in all patients suspected of having cardiac tumors.^[12]^ However, despite the advances in ultrasound imaging technologies and higher-frequency transducers, approximately 10% to 30% of 2-dimensional TTE remain inadequate.^[13]^ There are several entities that can mimic intracardiac tumors, including thrombi, mediastinal masses, metastatic lesions, vegetations, hiatal hernia, or variants of normal anatomy. Differentiation of benign from malignant cardiac masses is often challenging but important in clinical practice. Contrast-enhanced echocardiography has in recent years emerged as a modality to improve the diagnostic capacity of TTE for intracardiac masses, which can further improve morphologic assessment and perfusion evaluation can help differentiate the neovascularization of a malignant tumor from the avascularity of a thrombus and the sparse vascularity of a benign stromal tumor.^[14]^ Cardiac spindle cell sarcomas typically involve the left atrium and were often misdiagnosed as myxomas. Unlike myxomas, cardiac spindle cell sarcomas have a number of features, such as non-septal origin of mass, a broad attachment on the left atrial wall, semisolid consistency, especially high vascularization, and it should be hyper-enhanced on contrast echocardiography, as in our case. Then of course conventional 2-dimensional transthoracic echocardiography, combined with the contrast-enhanced echocardiography, which can delineate the perfusion of the cardiac mass, is useful for differentiation among types of suspected cardiac masses.^[15]^ In the present case, we used the contrast-assisted destruction-replenishment technique for tumor blood flow evaluation and detected quick contrast re-filling of the mass, thus also documented high vascularization of the malign tumors.

Ultrasound contrast agents have been shown to be safe. Dolan et al compared 23,659 patients from 3 U.S. medical centers who had received echo contrast with 5,900 controls who had not received contrast and found no increased mortality or nonfatal myocardial infarct in patients who had received contrast.^[16]^ In a recent meta-analysis of published studies on adverse cardiovascular events (myocardial infarction and all-cause mortality) with ultrasound contrast agents, the authors showed that there was no contrast agent-related increase in mortality or myocardial infarction. Adverse events associated with ultrasound contrast agents administration are mainly transient and mild, including nausea and vomiting, headache, flushing and rash.^[17]^ However, Solivetti et al reported the first case of severe anaphylactic shock induced by SonoVue (ultrasound contrast agents) in an individual with no clinically significant cardiac or pulmonary pathologies.^[18]^ Therefore, this possibility, although extremely rare, should be taken into account when ultrasound 2nd generation contrast medium SonoVue is administered.

Besides to echocardiography, CMR also provides useful information for characterizing cardiac masses. But the point in controversy is whether CMR can accurately distinguish benign from malignant lesions. Recent studies have illustrated that CMR has high accuracy for discriminating malignant versus benign masses, with a high rate of interobserver agreement.^[19]^ Chan et al clearly stated that late gadolinium enhancement CMR imaging enables cardiac neoplasm to be differentiated from thrombus based on vascular composition.^[20]^ Mousavi et al found both benign and malignant masses were commonly isointense on T1-weighted imaging, while benign masses were more often hyperintense and malignant masses demonstrated both hyperintense or isointense T2-weighted signals.^[21]^ Furthermore, first-pass perfusion of the mass was more frequent in malignant than benign tumors. Another study showed a pattern of hyperintensity/isointensity (compared with normal myocardium) with short T1 and hypo-intensity with long T1 was very frequent in thrombi (94%), rare in tumors (2%), and had the highest accuracy (95%) for the differentiation of both types of masses.^[19]^ Based on the results of the contrast-enhanced echocardiography and CMR, the mass in left atrium tended to be malignant in our case.

The main treatment method of cardiac primary tumor is surgical eradication of the mass in conjunction with adjuvant polychemotherapy, allowing 1 to achieve an increase of life expectancy compared to patients not operated on. Large complex left atrial masses may, however, present a considerable impediment to complete resection due to the posterior location of the left atrium and difficult accessibility.^[22]^ Cardiac autotransplantation with radical tumor resection is the treatment of choice for these cases. Cardiac autotransplantation (cardiac explantation, ex-vivo tumor resection, reconstruction, and reimplantation) was introduced for left-heart complex benign primary cardiac tumors by Cooley and for malignant tumors by Reardon. The autotransplantation approach has given us excellent visualization, which has enabled resection of the complete left atrium to achieve clear tumor margins. This method does not impose the necessity for a donor heart and it does not require subsequent immunosuppression.^[23]^ Previous studies reported the median survival among patients who underwent cardiac autotransplantation for primary cardiac sarcomas was 22 months, which was significant improved in comparison to the standard resection.^[24]^ Andrushchuk et al studied the cardiac autotransplantation as a method of surgical treatment for patients suffering primary massive malignant tumors of the left atrium. They strongly recommended only a total tumor resection allows prevention of tumor recurrence in the early and midterm period. They indicated that the intraoperative surgical strategy should be more radical with regard to resection of the left atrial wall, leaving the tumor resection margin more than 1 to 2 cm, and maximizing where possible the resected portion of the left atrium wall with the mitral valve and PV ostia. They were also prone to use adjuvant polychemotherapy (PCT) in this case, despite the role of adjuvant PCT and radiotherapy after pathomorphological assessment of the resected tissues was not fully studied.^[25]^ Our patient underwent the cardiac autotransplantation successfully, with subsequent adjuvant PCT, and the tumor has not recurred so far.

## 4 Conclusion

In summary, cardiac primary spindle cell sarcoma is very uncommon with nonspecific clinical and imaging characteristics according to limited case reports. The modern investigative methods including the contrast-enhanced echocardiography and CMR in the diagnosis of cardiac primary spindle cell sarcomas are of great value. And a multidisciplinary approach of surgery combined with PCT is an effective strategy for managing this disease.

## Author contributions

**Data curation:** Rui Li, Fei Ma, Haojie Li, Yujie Fei.

**Methodology:** Jin Qin, Zemin Fang.

**Resources:** Jin Qin, Haojie Li, Zemin Fang.

**Supervision:** Jin Qin.

**Writing – original draft:** Jin Qin.

**Writing – review & editing:** Jin Qin.

## Abbreviations

Abbreviations: CMR = cardiac magnetic resonance, CT = computed tomography, PCT = polychemotherapy, TTE = transthoracic echocardiography.
